# Epstein-Barr Virus-Positive Primary Central Nervous System Lymphoma in a 40-Year-Old Immunocompetent Patient

**DOI:** 10.7759/cureus.12754

**Published:** 2021-01-17

**Authors:** Sabastian Hajtovic, Cynthia Liu, Catherine M Diefenbach, Dimitris G Placantonakis

**Affiliations:** 1 Neurosurgery, City University of New York (CUNY) School of Medicine, New York, USA; 2 Pathology, New York University (NYU) Grossman School of Medicine, New York, USA; 3 Laura and Isaac Perlmutter Cancer Center, New York University (NYU) Grossman School of Medicine, New York, USA; 4 Neurosurgery, New York University (NYU) Grossman School of Medicine, New York, USA

**Keywords:** cns lymphoma, ebv, iummunocompetent

## Abstract

Epstein-Barr virus-positive (EBV+) primary central nervous system lymphoma (PCNSL) is a clinical entity rarely reported in young immunocompetent patients. Here, we present the case of a 40-year-old female with no history of immunosuppression or immunodeficiency, who presented with a ring-enhancing lesion in the right basal ganglia. The tumor generated significant vasogenic edema and mass effect, causing midline shift, symptoms of increased intracranial pressure, and rapidly progressive neurologic dysfunction. She underwent gross total resection of the tumor through a tubular retractor. Her tumor was of the diffuse large B cell lymphoma (DLBCL) subtype of PCNSL and was positive for EBV. No immunodeficiency or extracranial disease was identified. After adjuvant therapy with high-dose methotrexate, rituximab, and temozolomide, she remains disease-free two years after initial presentation. EBV+ PCNSL, although rare in young immunocompetent adults, poses unique clinical challenges and may require surgical intervention in the acute setting in some cases.

## Introduction

Primary CNS lymphoma (PCNSL) is a rare, highly aggressive extranodal subtype of non-Hodgkin’s lymphoma originating in the brain, spinal cord, eyes, or leptomeninges [1, 2]. PCNSL may represent 12-15% of lymphomas in HIV-AIDS, but only 1% of all lymphomas in the general population, and 4% of intracranial tumors in immunocompetent patients [1, 3, 4]. In the last decade, PCNSL has had a rising incidence in elderly, immunocompetent patients over the age of 60, with the highest incidence observed in those 70-79 years of age [1, 5].

PCNSL is classified histologically as diffuse large B-cell lymphoma (DLBCL) in 90-95% of cases, despite arising in the brain, which does not contain conventional lymphoid tissue [1-3, 6, 7]. The 2008 World Health Organization (WHO) classification of hematopoietic and lymphoid tissues considered PCNSL a distinct new subtype of DLBCL, characterized as an aggressive high-grade B-cell neoplasm with a poorer prognosis than its systemic counterpart [2, 7].

Epstein-Barr virus (EBV) is a gamma-herpesvirus found in over 90% of the general population. It is associated with multiple malignancies, including non-Hodgkin’s lymphomas and systemic AIDS-related B-cell lymphomas [8]. EBV positive (EBV+) PCSNL tumor cells are seen in nearly 100% of AIDS-related PCNSL, in which the EBV genome contributes to malignant transformation via expression of anti-apoptotic genes [3, 8, 9]. In immunocompetent hosts, an unregulated proliferation of EBV-infected lymphocytes is inhibited by cytotoxic T cells [3]. However, a decline in immunocompetence may result in activation of previously latent EBV and subsequent proliferation of B-cells [10]. Immunological deterioration with aging may explain the potential for EBV+ PCNSLs arising in otherwise immunocompetent patients over the age of 60 [4]. Importantly, however, EBV+ PCNSL remains very rare in immunocompetent patients, particularly in Western populations and patients under the age of 50 [8, 11, 12]. This is in contrast to AIDS-related PCNSL, which is more often seen in patients 20-60 years of age [3]. In addition, EBV+ PCNSLs have a worse prognosis than EBV-negative PCNSLs [8, 10]. 

Here, we present a rare case of EBV+ PCNSL, classified as DLBCL, in a young immunocompetent patient. We highlight the surgical and medical management, as well as the radiographic and histologic findings of the tumor, which make it a unique presentation in such a young patient.

## Case presentation

The patient is a 40-year-old, right-handed female with a past medical history of migraines, who presented in the emergency room with a persistent right-sided headache for one week. She characterized the headache as significantly more painful and different in quality from her previous migraines. She also reported gait instability, vomiting, and progressive lethargy since the onset of headache. Neurologic examination revealed lethargy and left-sided facial weakness, but no other focal deficits. The patient had no history of immunodeficiency, immunosuppressive medications, prior chemotherapy treatment, or clinical evidence of HIV infection or AIDS.

**Figure 1:**
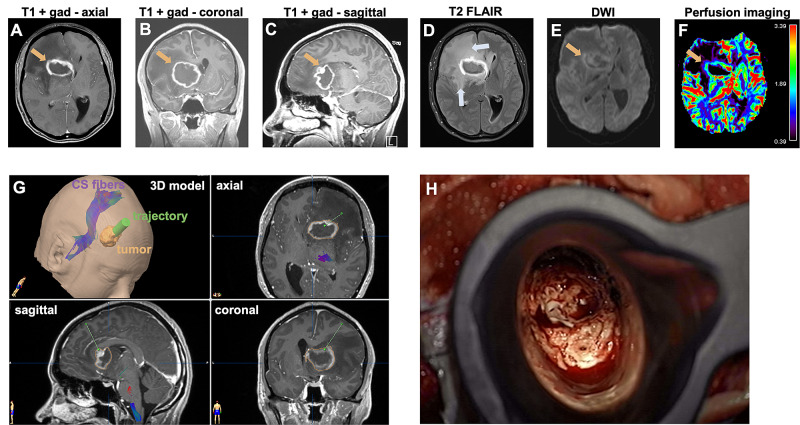
Preoperative imaging and surgical planning A, B, C: Preoperative T1-weighted MRI images enhanced with gadolinium show the ring-enhancing tumor (arrow) in the right basal ganglia. D: Axial T2 fluid-attenuated inversion recovery (FLAIR) image shows the peritumoral vasogenic edema (arrows). E: Axial diffusion-weighted image (DWI) shows only mild diffusion restriction (arrow). F: Axial perfusion imaging shows elevated relative cerebral blood flow (rCBV) along the enhancing rim of the tumor (arrow). G: Preoperative surgical planning on the BrainLab neuronavigation platform. The trajectory for placement of a tubular retractor is shown in green. Tractography was used to generate the corticospinal (CS) fiber tract. H: Intraoperative photograph showing the lesion visualized through the tubular retractor.

Magnetic resonance imaging (MRI) revealed a ring-enhancing tumor in the right basal ganglia, measuring approximately 3.9 x 2.9 x 3.6 cm (transverse by anteroposterior by craniocaudal dimension) (Figure 1A-1C). There was significant vasogenic edema (Figure 1D) associated with mass effect and a right-to-left subfalcine herniation of 9 mm. Compression of the ventricular system resulted in mild obstructive hydrocephalus. Diffusion-weighted imaging (DWI) showed mild restricted diffusion limited to the peripheral rim of the tumor (Figure 1E). Perfusion analysis demonstrated increased relative cerebral blood flow (rCBV) in the enhancing rim (Figure 1F).

Given the patient’s profound lethargy and radiographic signs of subfalcine herniation, we decided to perform an urgent surgical resection of the tumor. Under general anesthesia, we generated a right frontal craniotomy and accessed the tumor via a Viewsite Brain Access System (VBAS; Vycor Medical, FL, USA) tube (7 cm long, 17x11 mm wide) inserted through a trans-sulcal approach in the right middle frontal sulcus approximately 2 cm anterior to the coronal suture (Figure 1G-1H). The tumor extended to the ependyma of the right frontal horn of the ventricular system. An external ventricular drain (EVD) was left within the resection cavity, which communicated with the right lateral ventricle.

**Figure 2:**
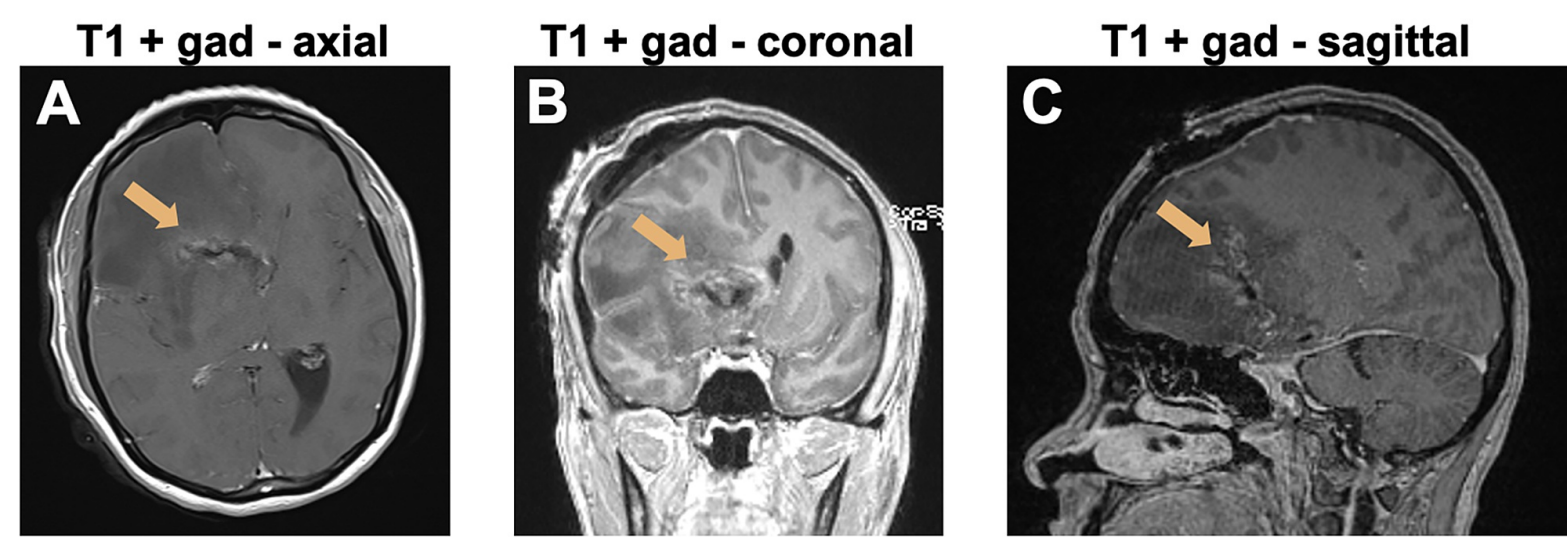
Postoperative MRI A, B, C: T1-weighted MRI with gadolinium enhancement on postoperative day 1 shows gross total resection of the enhancing mass (arrows).

**Figure 3:**
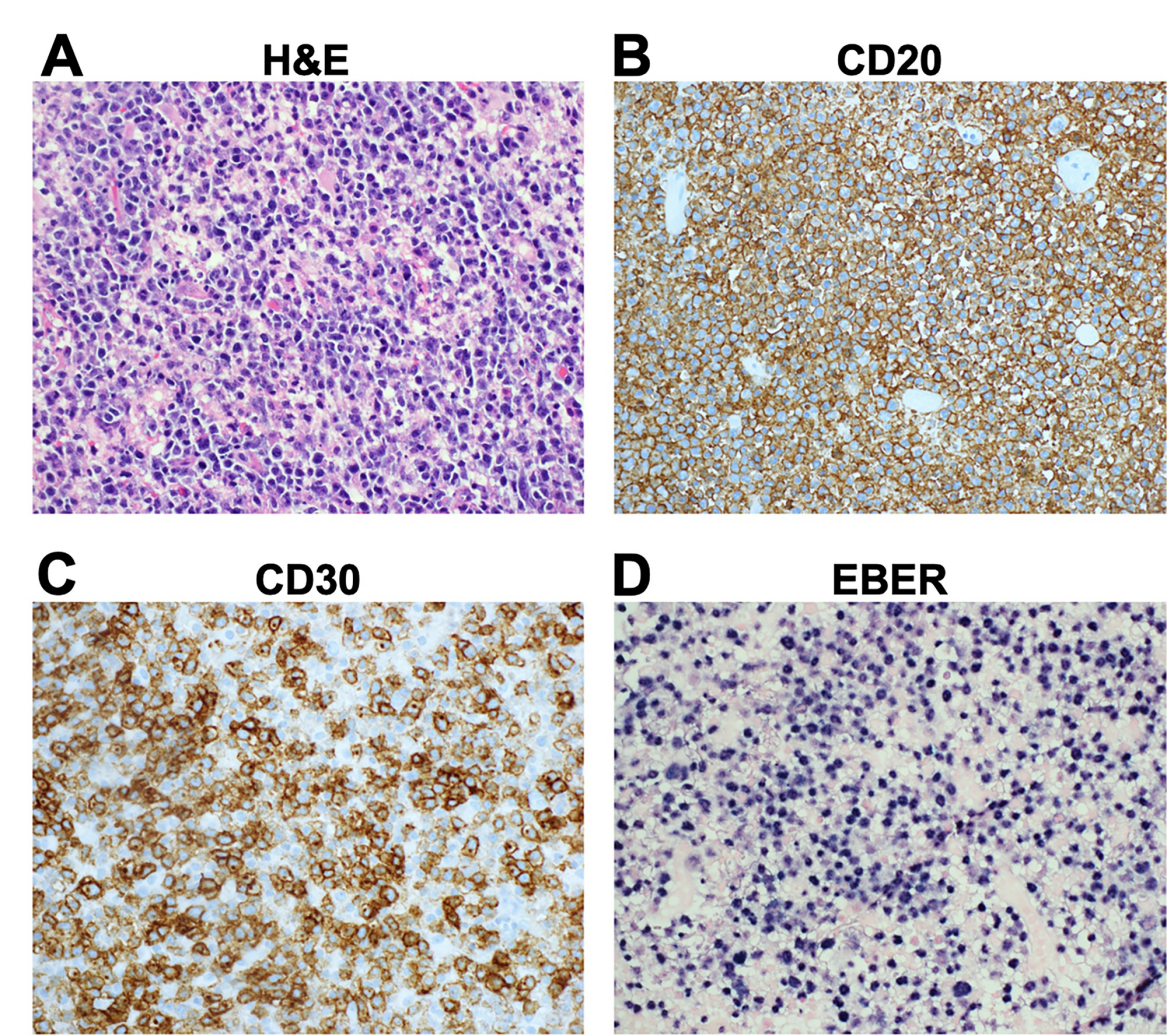
Histopathologic features Images were obtained at 400X magnification. A: On H&E stain, the large tumor cells were characterized by large nuclei, open chromatin, prominent nucleoli and moderate amounts of cytoplasm. B: Tumor cells were uniformly positive for CD20. C, D: The majority of tumor cells expressed CD30 (C) and were positive for Epstein-Barr virus-encoded small RNAs (EBER) by in situ hybridization (D).

Postoperatively, the patient’s mental status and the left facial weakness improved. Systemic steroid therapy was initiated. The EVD was removed on postoperative day seven. MRI on postoperative day one demonstrated gross total resection of the tumor (Figure 2A-2C). HIV testing resulted negative. Pathology was consistent with EBV+ DLBCL. Histologic examination revealed extensive necrosis and massive infiltration of large cells, with large nuclei, open chromatin, prominent nucleoli and moderate amounts of cytoplasm arranged in perivascular nodules (Figure 3A). Tumor cells were positive for CD20 (B-cell marker; Figure 3B), CD30 (Figure 3C), EBV-encoded small RNAs (EBERs) by in situ hybridization (ISH; Figure 3D), MUM-1, and PD-L1. CD10 was negative. There was rare BCL-2 and BCL-6 positivity. The Ki-67 proliferation index was 90%.

Additional workup was performed to rule out peripheral involvement. Computed tomography (CT) of the chest and abdomen demonstrated enlarged left axillary and subpectoral lymph nodes. Ultrasound-guided biopsy of the left axillary and cervical lymph nodes revealed benign lymphoid tissue. Bone marrow biopsy did not indicate peripheral disease. There was no ocular involvement. Flow cytometric analysis of cerebrospinal fluid from the EVD revealed paucicellular sample. Collectively, these findings suggested EBV+ PCNSL.

**Figure 4:**
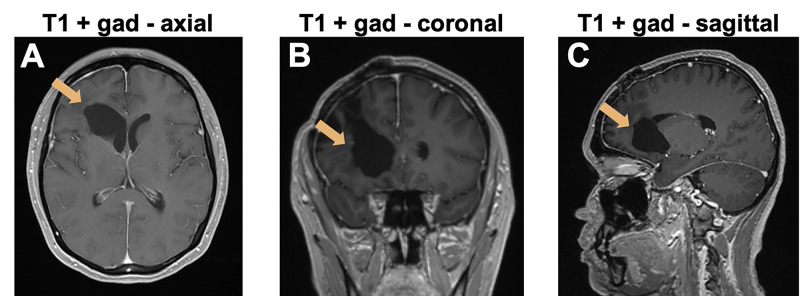
Delayed imaging two years after surgery A, B, C: T1-weighted MRI with gadolinium enhancement 25 months later shows no tumor recurrence. The arrows indicate the resection cavity.

She received adjuvant therapy with eight cycles of high-dose methotrexate and rituximab, followed by two cycles of high-dose cytarabine (HiDAC) consolidation. She then received four cycles of maintenance therapy with methotrexate. Because of the side-effects of methotrexate therapy, she then opted for maintenance therapy with temozolomide. She remains free of disease and neurologically intact 25 months after her initial diagnosis (Figure 4).

## Discussion

We present the rare case of a young immunocompetent patient who developed acute neurologic deterioration due to a large EBV+ PCNSL in the basal ganglia. Management included urgent surgical resection, and adjuvant therapy, which combined have led to sustained remission for over two years since the initial presentation. Multiple aspects of the case are unusual and merit discussion. First, EBV+ PCNSL in young immunocompetent patients is rare, especially in the United States. Second, the clinical deterioration was unusually rapid, suggesting a rapidly enlarging tumor. We postulate that the EBV positivity may have biologically contributed to rapid tumor cell proliferation, as demonstrated with the dramatically elevated Ki-67 proliferative index (90%) of the surgical specimen. Third, the diffusion restriction was limited, unlike most PCNSL cases, thus broadening the preoperative differential diagnosis to include primary brain tumor (high-grade glioma) and metastasis. Fourth, the rapid neurologic deterioration necessitated urgent surgical resection. This was achieved safely via a minimal access approach to the basal ganglia with a tubular retractor. Such retractors allow for resection of deep-seated tumors that have traditionally been considered inoperable. The surgical approach is, therefore of interest, not only as an essential component of therapy that generated the excellent clinical outcome in this case but also as a novel technique that warrants the attention of cranial surgeons.

Immunocompetent patients with PCNSL present most commonly with focal neurologic deficits, mental status changes, and symptoms of increased intracranial pressure (ICP) [1]. Our patient had these symptoms with a rapid onset of just one week. This is in contrast to the typical presentation in immunocompetent patients, in which symptoms develop progressively over weeks to months [1, 9]. In one review of 118 immunocompetent patients with PCNSL, Cheng et al. reported a median time from first symptoms to diagnosis of 28 days [13]. AIDS patients may develop PCNSL symptoms in just days, as our patient did, but these include constitutional symptoms [9], which our patient did not have.

In immunocompetent patients, PCNSLs have a deep, periventricular distribution, either solitary or multifocal [3, 5, 9, 14]. This characteristic distribution was seen in our case, in which the patient had a solitary lesion in the basal ganglia that extended to the ependyma of the right lateral ventricle. However, most immunocompetent PCNSL patients have homogenously enhancing tumors, whose DWI reveals robust restricted diffusion, while perfusion analysis reveals decreased rCBV due to increased cellular density and decreased vascularity [1, 5, 6, 9, 12]. Cheng et al. reported that ring enhancement was rarely seen in their cohort of immunocompetent PCNSL patients [13]. In contrast, ring enhancement occurs more commonly in AIDS-related PCNSL [3]. In our case, the tumor showed ring enhancement due to extensive central necrosis, increased rCBV, and only some diffusion restriction that was limited to the enhancing peripheral rim. Finally, immunocompetent PCNSL lesions are most often surrounded by moderate levels of edema and lack significant mass effect [5, 12]. However, our patient’s tumor was associated with extensive vasogenic edema contributing to subfalcine herniation and obstructive hydrocephalus.

Gene expression profiling studies of immunocompetent PCNSL have demonstrated molecularly heterogeneous tumors. The predominant DLBCL subtype is the non-germinal center, activated B-cell (ABC) phenotype, which is thought to contribute to a worse prognosis when compared to the germinal center phenotype [2, 7, 15]. The ABC phenotype is characterized by an almost universal expression of MUM1, a late germinal center marker [7], which was positive in our patient’s tumor. However, PCNSL may not align exactly with this particular subtype, as evidenced by ongoing germinal center exposure and other unique transcriptional features [6]. Some studies report 50-70% of patients with BCL-2 positive tumors, which may contribute to a poorer prognosis if expressed at high levels [2, 6, 15-17]. However, the percentage of BCL-2 positive tumor cells is known to vary [16]. In our patient, BCL-2 expression was low. CD30, positive in our patient’s tumor, has rarely been reported in the literature. One cohort of 75 immunocompetent patients found CD30 positivity in just one tumor (1.3%) [15]. PDL-1, which was also positive in our patient’s tumor, has been reported in 10.5% of immunocompetent cases [2].

EBV+ PCNSL may be seen in less than 5% of immunocompetent patients [11]. In fact, many studies report no patients with EBV+ tumors [2, 7, 14]. In a French cohort of 72 patients with PCNSL, EBV was not detected by EBER ISH in any of the non-Hodgkin’s lymphomas [18]. Other studies report small numbers of immunocompetent patients with EBV+ PCNSLs [4, 8, 10, 11, 15-17, 19, 20]. An Austrian cohort of 75 patients found just one EBV+ tumor (1.3%) [15]. In studies reporting the age of patients, the majority of EBV+ lesions are seen in patients over the age of 50 [4, 8, 10, 11, 17, 19, 20]. One Danish cohort of 41 patients found just two EBV+ cases (4.9%), both of which showed EBV positivity in less than 5% of tumor cells [16]. Rao et al. also report one such case displaying focal EBV positivity, a typical finding in immunocompetent cases, unlike AIDS-related PCNSL in which the majority of cells are positive [20]. This focal EBV positivity is in contrast to our patient, who had a tumor that was diffusely positive. Another cohort of 65 patients in India found EBV+ PCNSLs in just three of 64 DLBCLs (4.6%). One of these patients was a 21-year-old male whose tumor was of the germinal center subtype, making this a very rare case in the literature [17]. Similarly, another study reports a 36-year-old patient with an EBV+ PCNSL, but of the non-germinal center subtype [11]. Neither study reports the specific histologic or radiographic findings in these young patient’s tumors, thus not permitting further comparison of their features to our case.

A Turkish cohort of 32 patients found EBV+ PCNSLs in four patients (12.5%) [11]. The authors acknowledge that this percentage is higher than what is reported in Western countries for immunocompetent patients. Similarly, a Japanese study, including 21 immunocompetent patients, found two EBV+ cases (9.5%). The authors found no morphologic difference between the EBV+ and EBV negative tumors [8]. In contrast, another Japanese cohort of 57 patients found six EBV+ cases (10.5%) in patients over the age of 60, all of which had extensive necrosis, indicating that this feature may be more likely in immunocompetent patients if their tumors are EBV+ [4]. A third Japanese cohort of 33 patients found 16 cases (48%) with slight EBV positivity, ranging from 0.3 to 5.4% of tumor cells being positive. All 16 patients were at least 50 years old. There were also two patients (6.1%) with strongly EBV+ tumors, both over the age of 65. The authors attributed the unusually high percentage of slight EBV positivity to the much higher prevalence of latent EBV infection in Asia compared to Europe and the United States [10]. The significance of this finding remains uncertain. An important question to consider is whether EBV is the causative agent of PCNSL in these patients, as implicated in AIDS, or if it is simply a consequence of secondary activation due to local immunosuppression [11].

The standard of care in PCNSL treatment includes high dose methotrexate and rituximab as first-line induction therapy [5]. Traditionally, however, surgical resection has not been recommended due to diffusely infiltrative tumor growth and a lack of therapeutic benefit [1, 5, 6]. This view was challenged by a secondary analysis of a large German cohort of PCNSL patients, in which there was improved progression-free survival with total or subtotal tumor resection [6]. Bellinzona et al. also reported that surgical resection, when combined with chemo- and/or radiotherapy, prolonged survival in a subset of patients. However, unlike other studies, over two-thirds of their cohort presented with signs of increased ICP and progressive neurologic deficits [14]. This made emergent surgical intervention an essential part of treatment. These symptoms were seen in our case as well, in which tumor resection followed by the standard chemotherapy regimen had a very favorable outcome for the patient.

## Conclusions

EBV+ PCNSL is a distinct clinical entity that is rarely reported in immunocompetent patients, especially in Western countries and in patients under the age of 50. Studies of EBV+ PCNSL in immunocompetent patients do not consistently report clinical, radiographic, and histologic findings. We present a rare case of EBV+ PCNSL in a 40-year-old immunocompetent patient who had a large, rapidly growing tumor in the basal ganglia causing rapid neurologic deterioration. The acute presentation prompted urgent surgical resection of the tumor with a tubular retractor, followed by a standard regimen of methotrexate and rituximab, with a favorable outcome. It is important for neurosurgeons and other clinicians to be aware of the potential for such a unique clinical presentation of EBV+ PCNSL in young immunocompetent patients. It is also important for cranial surgeons to consider tubular retractor approaches to deep-seated brain tumors if needed.
